# Community Pharmacists’ Perceptions of Their Role in Provision of Anemia Management in Jazan Region, Saudi Arabia, and the Associated Barriers

**DOI:** 10.3390/healthcare10081452

**Published:** 2022-08-02

**Authors:** Osama A. Madkhali, Fahad Alzahrani

**Affiliations:** 1 Department of Pharmaceutics, College of Pharmacy, Jazan University, Jazan 45124, Saudi Arabia; 2 Clinical and Hospital Pharmacy Department, College of Pharmacy, Taibah University, Madinah 42353, Saudi Arabia; fzahrani@taibahu.edu.sa

**Keywords:** anemia management, perceived practice, barriers, community pharmacy, perceptions

## Abstract

Background: As a result of the lack of research exploring community pharmacists’ perspectives on anemia care, this study examined the perceived practices and barriers to anemia management services in Saudi Arabia. Methods: A questionnaire was administered to community pharmacists to validate pharmacist perceptions of their role in anemia care. Using an 11-item role test, pharmacists were asked about their perceptions of anemia management. Pharmacy anemia management barriers were explored using 20 items, and their perceptions of inter-professional contact were examined by two items. Data analyses were performed using SPSS version 22. Results: This research involved 324 community pharmacists, 62.3% of whom were males. There were significant differences between the location of pharmacy education and the perceived practice of anemia management. The most common barriers to anemia counseling were patients’ lack of knowledge about anemia, health beliefs, patients’ perceptions that their doctor takes care of them, and time constraints. The majority of respondents said they would like to have more contact with other healthcare professionals regarding the care of anemia patients. Conclusions: A positive perception of pharmacists’ role in anemia management is prevalent among Saudi pharmacists in the Jazan region. Anemia management is challenging due to time limitations and patient-related problems.

## 1. Introduction

Anemia is a blood illness marked by a lack of healthy red blood cells capable of transporting enough oxygen to the tissues [1]. It affects a large number of people around the world, especially in developing countries, resulting in a huge financial burden due to the high cost of medical care [2]. The World Health Organization (WHO) has revealed that anemia affects nearly a quarter of the world’s population, particularly newborns and women [3]. The prevalence of anemia in the Middle East (25 to 35%) is comparable to that in underdeveloped nations [4,5], in contrast to 5–8% prevalence in developed countries.

It has been reported that anemia is prevalent in 30–56% of Saudi Arabians [6]. Due to factors such as consanguineous marriages among first-degree families, lack of effective screening programs, and malaria epidemics, the Jazan region in the southern region of Saudi Arabia is one of the areas most affected by a group of inherited red blood cell disorders, particularly sickle cell anemia (SCA) [4]. Anemia is characterized by symptoms such as severe pain, acute chest syndrome, cerebrovascular accidents, recurring infections, hypoxia, and growth impairment [7]. These symptoms necessitate timely treatment and cooperation from all health specialists, including pharmacists.

Several studies have shown the role of healthcare specialists in the management of many chronic diseases [8,9,10,11,12]. Pharmacists have demonstrated a good impact on the management of different diseases such as diabetes [13], hypertension [14], and asthma [15]. Since anemia is a symptom-related disease, anemia management is very significant for either the patients themselves or the healthcare specialists. Early detection and treatment of the symptoms help in reducing the risk of morbidity and mortality, eliminating the complications of anemia, including frequent hospitalizations or allogeneic blood transfusion, and reducing cost [16,17,18,19]. Therefore, the role of healthcare community is to improve the quality of life of anemic patients by creating a shared quality improvement program that helps in all aspects of anemia management. Community pharmacists have significant participation in anemia management due to the frequent visit of anemic patients to the pharmacy.

In recent years, the scope of the practice of community pharmacists has increased appreciably to include the provision of services aimed at improving health outcomes [20]. Arising from their expertise in drugs, constant interaction with patients, and easy accessibility, pharmacists are in a unique position to assist patients in managing chronic illnesses [7,20,21]. Ironically, pharmacists are frequently underutilized in the provision of improved anemia management in primary care [22]. Community pharmacies represent the initial point of contact for many people who require healthcare in poor and underdeveloped nations [23]. Therefore, community pharmacists play a critical role in the treatment of chronic illnesses, an example of which is anemia.

To date, no research has been conducted to assess the perception of community pharmacists regarding their potential role in anemia management in the Jazan region of Saudi Arabia. As a result, the purpose of this study was to assess community pharmacists’ perceptions of anemia management practices and barriers.

## 2. Materials and Methods

### 2.1. Study Design and Eligibility Criteria

From October to December 2020, a descriptive cross-sectional study was conducted among community pharmacists in the Jazan region of Saudi Arabia. All full licensed Saudi Arabian pharmacists working in community pharmacies in the Jazan region with a bachelor’s degree or higher were eligible to participate in this study. In 2017, approximately 600 pharmacists practiced in the Jazan region of Saudi Arabia [24]. Using Raosfot^®^’s online sample size calculator, it was estimated that the minimum effective sample size in this study was 234, with a 95% confidence interval and a 5% error margin.

### 2.2. Sampling Procedure

Community pharmacists were recruited using convenience sampling. Six trained research assistants presented the study design and objectives to the community pharmacists. A link to the questionnaire was given to pharmacists who agreed to participate in the study, and they were asked to complete the questionnaire while the research assistants were on duty. Using this method, any issues raised during data collection were addressed consistently so as to enhance the degree of response. For pharmacists who were not seen during the first visit due to other engagements, the research assistants were asked to schedule another appointment at their convenience so that the study could be continued at a later date.

### 2.3. Data Collection

The responses were collected using a structured, self-administered questionnaire. The questionnaire used for this study was developed and modified based on the literature review [25,26,27,28,29,30] and the experience of the investigators (Table 1). The self-administered questionnaire comprised four sections. In the first section (demographics), the community pharmacists were required to provide information on nine demographic questions: age, gender, level of education, place where pharmacy degree was obtained, number of years of practice as licensed pharmacists in Saudi Arabia, degree of interest of pharmacists in anemia management, history of attendance/participation in structured or organized continuing education programs on anemia management, and estimates of the number of anemia patients they interacted with each week.

In the second part of the survey (role), 11 questions were posed to the community pharmacists who responded on a five-point Likert scale ranging from 1 (strongly disagree) to 5 (strongly agree). Each item was rated 4 or 5, depending on how well it was received.

In the third section (barriers), there were 20 barriers that militated against the provision of pharmacy anemia management services. A five-point Likert scale was used to assess how these barriers influenced the ability of community pharmacists to provide specific anemia counseling or services. The score on the five-point Likert scale ranged from 1 (no impact) to 5 (high impact). The last section of the questionnaire was based on inter-professional contact, and responses were ranked on a five-point Likert scale (1 = strongly disagree, 5 = strongly agree).

Two faculty members in the College of Pharmacy at Jazan University evaluated and reviewed the validity of the questionnaire items. The survey items were rated according to their relevance on a scale of 1–5, with 1 indicating that the item was not relevant, and 5 indicated that the item was highly relevant. The reviewers decided a priori to include in the final questionnaire only those statements rated as pertinent or highly relevant. Disputes in ratings between the two reviewers were resolved through discussion and compromise. A pilot test (*n* = 7) was used to evaluate the clarity and comprehension of the items included in the questionnaire. Pilot group comments and feedback led to minor changes in the study tool. These changes were intended to enhance the clarity of the survey items. The pilot sample data were excluded from the final analysis. Cronbach’s alpha (an index of reliability) was evaluated for internal consistency. A Cronbach’s alpha of more than 70% was taken as indicative of internal consistency [31].

### 2.4. Statistical Analysis

The data obtained in this study were analyzed using the IBM Statistical Package for the Social Sciences (SPSS Inc., Chicago, IL, USA) version 22. Data were analyzed using descriptive statistics such as percentages, means, and standard deviations. The Shapiro–Wilk tests were used to assess scores for normality of distribution. In the absence of normal distribution, the Mann–Whitney test and Kruskal–Wallis test were used. Values of *p* = 0.05 were considered indicative of statistically significant differences. All *p* values were two-sided.

### 2.5. Ethical Approval

Approval for this study was received from the Human Research Ethics Committee of Taibah University in Madinah, Saudi Arabia (COPTU-REC-22; 24 September 2021). All pharmacists involved in this research signed informed consent prior to their enrollment.

## 3. Results

### 3.1. Internal Consistencies

The study questionnaire items were internally consistent, as indicated by an overall Cronbach’s alpha value of 83.9%. Cronbach’s alpha values were also computed separately for each of the domains in the study. The values of Cronbach’s alpha for 11 roles and current practice, 20 barrier items, and 2 inter-professional contacts were 96.8, 92.6, and 50%, respectively.

### 3.2. Demographics

In this study, 324 out of 600 community pharmacists were recruited in the Jazan region, Saudi Arabia. This met the sample size requirement, and the resultant response was 54.6%. The ages of the community pharmacists ranged from 21 to 60 years, with a mean age of 29.53 years. The majority of participants (62.3%) were males, and most of them (75.5%) hold PharmD. More than half of the participants (56.8%) graduated from the University of Jazan. The average years of pharmacy practice (experience) ranged from 0 to 12, while the average years of experience as licensed pharmacists in Saudi Arabia ranged from 0 to 10. Almost half of them (*n* = 268) indicated an interest in counseling anemia patients, despite the fact that most pharmacists (*n* = 204) had never participated in continuing education programs related to anemia management. Table 2 shows other demographic and pharmacy practice characteristics.

### 3.3. Community Pharmacists’ Perceptions of Their Role in Anemia Management

More than half of the studied pharmacists (74.4%) believed that community pharmacists play an important role in counseling anemia patients. Approximately 21% of pharmacists (*n* = 71) strongly agreed that pharmacists should be interested in patient’s drug history comprising prescriptions, OTC, herbal products, and natural products. Moreover, 24.1% of the pharmacists (*n* = 78) strongly agreed that pharmacists should explain to anemic patients the potential adverse effects of iron supplements. The majority of the pharmacists (20.4%; *n* = 66) strongly agreed that anemic patients should receive counseling on medications that may worsen anemic conditions, while 71 pharmacists (20.4%) strongly agreed on counseling on signs and symptoms of anemia. However, 92 pharmacists (28.4%) were neutral on self-management of anemia. There was almost a unanimous agreement among 29.3% of the pharmacists that part of their role was to refer patients who required medical attention to their pharmacies. The detailed responses of the pharmacists are provided in Table 3.

The Kruskal–Wallis H test was applied to compare the mean differences in perceived practice scores among different groups with respect to age, level of education, the place from where their academic degree was obtained, number of years of practice as a pharmacist, number of years of practice as a licensed pharmacist in Saudi Arabia, and number of patients with anemia seen in a typical week. The Mann–Whitney U test was applied to compare mean differences in gender and attendance of continuing education events related to anemia management. These results are presented in Table 4. There was a significant relationship between pharmacy educational sources and perceptions of anemia management. Pharmacists who graduated from foreign universities had a more positive perception of their practice than pharmacists who graduated from Saudi universities. However, there was no significant relationship between respondents’ age, gender, ethnicity, and duration of practice.

More than half of the participants indicated that each of the 20 potential barriers had some impact. The pharmacists identified the four most common barriers which impacted their ability to provide anemia services. These comprised a lack of awareness of anemia by patients, health beliefs of patients, patients’ confidence in the care being provided by their doctors, and time constraints on the part of the patients. In terms of patient-related factors, language barriers and the patient’s perception of the pharmacist’s role were two factors that posed the greatest obstacles. In contrast, the pharmacists indicated that lack of financial incentive and conflict between professional and commercial interests did not have significant impacts on their ability to provide specific anemia services. The barriers identified as potential obstacles to anemia management by community pharmacists are shown in Table 5 and Table 6.

### 3.4. Community Pharmacists’ Perceived Level of Inter-Professional Contact

Approximately 36.1% of community pharmacists stated that they had good inter-professional contact when caring for patients with anemia, while 57.1% of the pharmacists said they would like to have more inter-professional contacts. Details of these responses are shown in Table 7.

## 4. Discussion

With a score of 70.8 ± 1.1, it is evident that community pharmacists had a positive perception of their role in anemia care. Pharmacists perceived that their role in anemia management involved three major components: patient self-management, medication use, and anemia control. This is consistent with extant literature showing positive perceptions regarding the role of community pharmacists in the management of chronic diseases [32,33,34]. Furthermore, our results indicated that the guidelines for managing anemic patients did not stipulate the role of a typical community pharmacist in Jazan, Saudi Arabia. Thus, the perception of community pharmacists about their role might be of prime importance in the future management of anemia.

More than half of the community pharmacists believed that they should provide advice to patients on how to monitor, control, and manage anemia. It is important for patients to understand the timing of administration of oral iron supplements, as well as the potential adverse effects of iron supplements and medications which may exacerbate anemia. On average, 58% of the pharmacists (*n* = 188) perceived the need to assist patients with selecting effective and efficient supplements.

A significant number of community pharmacists (198; 61%) counseled patients on drug–drug interactions, while 54.8% of the pharmacists (*n* = 178) gave advice on diets in connection with anemia management. However, only 161 of the community pharmacists (49.6%) indicated that they counseled patients on exercise in connection with anemia management. Similar findings were reported in previous studies [25,27].

This study observed a significant difference between pharmacy educational sources and perceived anemia management practices. Those pharmacists who graduated from foreign universities had a more positive perception of their practice than those who graduated from Saudi universities. A possible explanation for this result is that most pharmacy schools’ curriculums in Saudi Arabia do not prepare graduates well enough to provide chronic disease management services, including anemia. Alaqeel and Abanmy found that many Saudi pharmacy schools do not offer any training in a community pharmacy before graduation [35]. In addition, pharmacy interns are currently limited to filling prescriptions in a community pharmacy without anything to do with counseling or providing other services [36].

The study found that both community pharmacists and anemic patients encountered significant and common barriers to anemia counseling. In terms of factors related to community pharmacists, 35% or more indicated that the most significant barriers to anemia counseling were insufficient time, difficulty in knowing patients’ needs, lack of counseling space, and the lack of government or employer support. Patients’ lack of time, the perception that it is not the role of pharmacists to treat anemia, health beliefs, lack of knowledge about anemia, and the patient’s confidence in the care being provided by doctors, were the most prominent barriers related to anemia counseling. Similar types of barriers were identified in previous studies [25,27,37]. Furthermore, 204 (63%) of the community pharmacists expressed interest in participating in continuing education programs related to anemia management. In order to improve anemia management and enhance health care outcomes in the future, it is necessary to develop an anemia educational care program to enhance pharmacists’ knowledge and awareness about anemia and also provide patient counseling points for pharmacists to consider when discussing anemia [38].

Moreover, this study investigated the expectations of pharmacists regarding their inter-professional relationships in light of the fact that international and national guidelines for anemia management advocate a multidisciplinary approach. In spite of the fact that most participants had contact with other health care professionals regarding the care of anemic patients, almost 57% of the pharmacists would like to have more of these interactions. It is clear from existing literature that inter-professional relationships are critical to anemia management [27]. Although the current study did not investigate this issue further, the strength of the responses to this question, and the identification of strong barriers militating against the perceived roles of physicians and pharmacists, suggest the need for more studies in this area.

This study has several strengths, such as a high response rate from recruited respondents and internal consistency in responses, as measured by Cronbach’s alpha. However, the study has some limitations. In the first place, it was conducted only among pharmacists in one region of Saudi Arabia (Jazan). Thus, the findings of this study cannot be applied to the entire population of community pharmacists in Saudi Arabia. Secondly, since the study was cross-sectional, the findings obtained are only correlational, not causal. Lastly, the questionnaire was developed based on current anemia management guidelines, previous research, and expert opinion, without qualitative research.

## 5. Conclusions

The pharmacists had a positive perspective on improved management of anemia. Patients were perceived to have a three-dimensional role in anemia care; these comprised self-management, medication use, and anemia control. Furthermore, this study has identified barriers to anemia counseling from the perspective of both pharmacists and patients. There were several barriers to providing anemia services, such as time and patient concerns. Research should be conducted in the future to determine barriers to the expansion of community pharmacists’ roles in anemia management, given the current international push to manage chronic diseases in primary care and the evidence that pharmacy-based disease state management services could benefit patients.

## Figures and Tables

**Table 1:** The 33-item community pharmacist’s role in anemia management questionnaire.

**Section 1 (Role): The Pharmacists’ Perception of Their Role Towards Anemia Management**
Please indicate your level of agreement on each of the following statements about the role of a pharmacist when dealing with an anemic patient: Conduct a drug history, including prescription medications, OTC, Herbal, and natural products Describe the appropriate time to administer oral iron supplements Describe potential adverse effects of iron supplements Assist with food-food and food-drug interaction Assist with the selection of more efficient supplements Provide basic information on diet as it relates to anemia management Provide basic information on exercise as it relates to anemia management Counsel on medications that can exacerbate conditions Counsel on signs and symptoms of anemia Anemia self-management by the patient (i.e., recognizing when and knowing how to take action when anemia gets worse) Refer patients who need to seek medical attention
**Section 2 (Barrier): Barriers that Prevent Pharmacists from Providing Anemia Healthcare**
Please indicate to what extent you feel each of the following factors impacts the pharmacist’s ability to provide specific anemia counseling or services: Lack of time by the pharmacist Lack of time by the patient Pharmacists’ perception that it is not their role Patient’s perception that it is not the pharmacist’s role Language barriers Patient’s health beliefs Patient’s lack of anemia knowledge Patient perception that they are already well cared for by the doctor Conflict between professional and commercial interests Trying not to ‘overstep’ the role of the doctor No financial incentive Difficulty to know patient’s needs Lack of counseling space Lack of governmental or employer support
**Lack of Confidence or skills in:**
15. Anemia medication counseling 16. Anemia adherence counseling 17. Anemia self-management counseling 18. Anemia trigger factor counseling 19. Reviewing and counseling about anemia control 20. Anemia monitoring
**Section 3 (Inter-professional contact):**
Please indicate your level of agreement with each of the following statements: I have good interprofessional contact with other healthcare professionals with regard to care of my patients with anemia I would like to have more contact with other healthcare professionals with regards to the care of my patients with anemia

**Table 2:** Demographic and practice characteristics.

Characteristics	*n* (%)
**Age**	
21–29	202 (62.3)
30–39	101 (31.2)
40–49	20 (6.2)
50–60	1 (.3)
**Gender**	
Male	201 (62.3)
Female	122 (37.7)
**Years as a pharmacist**	
0–2	146 (45.1)
3–5	90 (27.8)
6–10	64 (19.8)
>10	24 (7.4)
**Years as a licensed pharmacist in Saudi Arabia**	
0–2	156 (48.1)
3–5	94 (29.0)
6–10	60 (18.5)
>10	14 (4.3)
**Level of Education**	
BSc.	78 (24.1)
PharmD	244 (75.5)
MSc	2 (0.6)
**Source of Education**	
University of Jazan	184 (56.8)
Other Saudi Universities	55 (17)
Foreign University	85 (26.2)
**Interesting in counseling Anemia**	
Yes	268 (82.71)
No	56 (17.31)
**Attendance of Continuing Education Events Related to Anemia Management**	
Yes	204 (63.00)
No	120 (37.00)
**Number of Patients with Anemia seen in a Typical Week**	
0–5	234 (72.23)
6 to 10	61 (18.81)
11 to 20	18 (5.61)
More than 20	11 (3.4)

**Table 3:** Perceived roles of pharmacists in counseling patients with anemia.

Item Number	Items	Strongly Disagree *n* (%)	Disagree *n* (%)	Neutral *n* (%)	Agree *n* (%)	Strongly Agree *n* (%)
1	Conduct a drug history, including prescription medications, OTC, Herbal, and natural products.	23 (7.1)	29 (9.0)	67 (20.7)	134 (41.4)	71 (21.9)
2	Describe the appropriate time to administer oral iron supplements.	22 (6.8)	27 (8.3)	71 (21.9)	128 (39.5)	76 (23.6)
3	Describe potential adverse effects of iron supplements.	26 (8.0)	28 (8.6)	62 (19.1)	130 (40.1)	78 (24.1)
4	Assist with drug-drug and food-drug interaction	21 (6.5)	28 (8.6)	77 (23.8)	115 (35.5)	83 (25.6)
5	Assist with the selection of more efficient supplements.	25 (7.7)	32 (9.9)	79 (24.4)	123 (38.0)	65 (20.1)
6	Provide basic information on diet as it relates to anemia management.	31 (9.6)	41 (12.7)	74 (22.8)	115 (35.5)	63 (19.3)
7	Provide basic information on exercise as it relates to anemia management.	25 (7.7)	50 (15.4)	88 (27.2)	109 (33.6)	52 (16.0)
8	Counsel on medications that can exacerbate conditions.	25 (7.7)	29 (9.0)	79 (24.4)	118 (36.4)	66 (20.4)
9	Counsel on signs and symptoms of anemia	27 (8.3)	29 (9.0)	79 (24.4)	118 (36.4)	71 (21.9)
10	Anemia self-management by the patient.	25 (7.7)	33 (10.2)	92 (28.4)	116 (35.8)	58 (17.9)
11	Refer patients who need to seek medical attention	27 (8.3)	32 (9.9)	61 (18.8)	109 (33.6)	95 (29.3)

**Table 4:** Distribution of perceived practice of anemia according to socio-demographic variables.

Variables	Groups	*n*	Mean ± SD	*p*-Value
Age	21–29	202	3.46 ± 1.11	0.73
	30–39	101	3.71 ± 0.82	
	40–49	20	3.63 ± 0.85	
	50–60	1	3.00 ± -	
Gender	Male	201	3.61 ± 0.97	0.13
	Female	122	3.44 ± 1.06	
Years as a pharmacist	0–2	146	3.38 ± 1.10	0.10
	3–5	90	3.74 ± 0.91	
	6–10	64	3.72 ± 0.82	
	>10	24	3.31 ± 1.11	
Years as a licensed pharmacist in SA	0–2	156	3.43 ± 1.10	0.45
	3–5	94	3.71 ± 0.90	
	6–10	60	3.54 ± 0.98	
	>10	14	3.67 ± 0.79	
Level of education	BSc.	78	3.75 ± 089	0.12
	PharmD	244	3.48 ± 1.01	
	MSc	2	3.36 ± 0.77	
Source of education	University of Jazan	184	3.47 ± 1.09	0.04
	Other Saudi Universities	55	3.35 ± 0.97	
	Foreign University	85	3.83 ± 0.76	
Interesting in counseling anemia	Yes	268	3.57 ± 1.01	0.20
	No	56	3.41 ± 1.00	
Attendance of continuing education events related to anemia management	Yes	204	3.61 ± 103	0.32
	No	120	3.43 ± 0.96	
No. of patients with anemia seen in a typical week	0–5	234		
	6–10	61		
	11–20	18		
	>20	11		

**Table 5:** Perceived practice barriers to pharmacist’s ability to provide specific anemia counseling or service.

Item Number	Item	No Impact *n* (%)	Slight Impact *n* (%)	Moderate Impact *n* (%)	Considerable Impact *n* (%)	High Impact *n* (%)
1	Lack of time by the pharmacist	41 (12.7)	42 (13.0)	123 (38.0)	71 (21.9)	47 (14.5)
2	Lack of time by the patient	32 (9.9)	51 (15.7)	104 (32.1)	52 (16.0)	85 (26.2)
3	Pharmacists’ perception that it is not their role.	47 (14.5)	56 (17.3)	110 (34.0)	38 (11.7)	73 (22.5)
4	Patient’s perception that it is not the pharmacist’s role	43 (13.3)	50 (15.4)	111 (34.3)	71 (21.9)	49 (15.1)
5	Language barriers	86 (26.5)	55 (17.0)	85 (26.2)	66 (20.4)	32 (9.9)
6	Patient’s health beliefs	29 (9.0)	56 (17.3)	113 (34.9)	75 (23.1)	51 (15.7)
7	Patient’s lack of anemia knowledge	25 (7.7)	44 (13.6)	121 (37.3)	67 (20.7)	67 (20.7
8	Patient perception that they are already well cared for by the doctor	30 (9.3)	38 (11.7)	103 (31.8)	82 (25.3)	71 (21.9)
9	The conflict between professional and commercial interests	51 (15.7)	57 (17.6)	115 (35.5)	63 (19.4)	38 (11.7)
10	Trying not to ‘overstep’ the role of the doctor	38 (11.7)	62 (19.1)	115 (35.5)	75 (23.1)	34 (10.5)
11	No financial incentive	56 (17.3)	63 (19.4)	109 (33.6)	63 (19.4)	33 (10.2)
12	Difficulty to know patient’s needs	42 (13.0)	57 (17.6)	110 (34.0)	80 (24.7)	35 (10.8)
13	Lack of counseling space	47 (14.5)	51 (15.7)	99 (30.6)	83 (25.6)	44 (13.6)
14	Lack of governmental or employer support	62 (19.1)	45 (13.9)	96 (29.6)	70 (21.6)	51 (15.7)

**Table 6:** Lack of confidence or skills.

Item Number	Items	No Impact *n* (%)	Slight Impact *n* (%)	Moderate Impact *n* (%)	Considerable Impact *n* (%)	High Impact *n* (%)
1	Anemia medication counseling	72 (22.2)	87 (26.9)	81 (25.0)	64 (19.8)	20 (6.2)
2	Anemia adherence counseling	62 (19.1)	93 (28.7)	91 (28.1)	59 (18.2)	19 (5.9)
3	Anemia self-management counseling	62 (19.1)	94 (29.0)	85 (26.2)	65 (20.1)	18 (5.6)
4	Anemia trigger factor counseling	54 (16.7)	79 (24.4)	107 (33.0)	66 (20.4)	18 (5.6)
5	Reviewing and counseling about anemia control	61 (18.8)	79 (24.4)	98 (30.2)	65 (20.1)	21 (6.5)
6	Anemia monitoring	59 (18.2)	83 (25.6)	90 (27.8)	67 (20.7)	25 (7.7)

**Table 7:** Inter-professional contact.

Item Number	Item	Strongly Disagree *n* (%)	Disagree *n* (%)	Neutral *n* (%)	Agree *n* (%)	Strongly Agree *n* (%)
1	I have good interprofessional contact with other healthcare professionals with regard to the care of my patients with anemia.	55 (17.0)	57 (17.36)	95 (29.3)	83 (25.6)	34 (10.5)
2	I would like to have more contact with other healthcare professionals with regard to the care of my patients with anemia.	23 (7.1)	30 (9.3)	84 (25.9)	89 (27.5)	96 (29.6)

## Data Availability

Data are available upon reasonable request by contacting the corresponding author.
